# Crystallography at the nanoscale: planar defects in ZnO nanospikes

**DOI:** 10.1107/S1600576719009415

**Published:** 2019-08-29

**Authors:** Niklas Wolff, Viktor Hrkac, Jeffrey J. Ditto, Viola Duppel, Yogendra K. Mishra, David C. Johnson, Rainer Adelung, Lorenz Kienle

**Affiliations:** a Synthesis and Real Structure and Institute for Material Science, Kiel University, Kaiserstrasse 2, Kiel 24143, Germany; b Department of Chemistry and Biochemistry and Materials Science Institute, University of Oregon, Eugene, OR 97403, USA; c Nanochemistry, Max Planck Institute for Solid State Research, Heisenbergstrasse 1, Stuttgart 70569, Germany; d Functional Nanomaterials and Institute for Material Science, Kiel University, Kaiserstrasse 2, Kiel 24143, Germany

**Keywords:** cross-section specimen preparation, high-resolution transmission electron microscopy, 3D defect reconstruction, anisotropic nanostructures

## Abstract

The examination of anisotropic nanostructures, such as wires, platelets or spikes, inside a transmission electron microscope is normally performed only in plan view. However, intrinsic defects such as growth twin interfaces could occasionally be concealed from direct observation for geometric reasons, leading to superposition. This article presents the shadow-focused ion-beam technique to prepare multiple electron-beam-transparent cross-section specimens of ZnO nanospikes, via a procedure which could be readily extended to other anisotropic structures. In contrast with plan-view data of the same nanospikes, here the viewing direction allows the examination of defects without superposition. By this method, the coexistence of two twin configurations inside the wurtzite-type structure is observed, namely  and , which were not identified during the plan-view observations owing to superposition of the domains. The defect arrangement could be the result of coalescence twinning of crystalline nuclei formed on the partially molten Zn substrate during the flame-transport synthesis. Three-dimensional defect models of the twin interface structures have been derived and are correlated with the plan-view investigations by simulation.

## Abstract 2

A cross-section analysis is presented of defects in ZnO nanospikes, investigated by means of transmission electron microscopy and defect simulation.

## 1. Introduction   

Nanostructured zinc oxide (ZnO) semiconductors have attracted much research interest over recent decades owing to their diversity of chemical and physical properties (Özgür *et al.*, 2005 ▸; Meyer *et al.*, 2004 ▸; Pearton, 2005 ▸; Yang *et al.*, 2002 ▸; Djurišić & Leung, 2006 ▸; Li *et al.*, 2008 ▸). Due to the characteristic of a wurtzite-type structure (denoted by a superscript W; space group *P*6_3_ *mc*), *i.e.* the absence of inversion symmetry, the piezoelectric effect is present in ZnO. With a direct bandgap of 3.37 eV and an exciton binding energy of 60 meV at room temperature, ZnO nanostructures are promoted for applications in the fields of electronic materials (Jebril *et al.*, 2010 ▸; Gupta, 1990 ▸), opto-electronics (Keis *et al.*, 2002 ▸; Könenkamp *et al.*, 2000 ▸), sensor devices (Liu *et al.*, 2016 ▸; Chai *et al.*, 2012 ▸; Lupan *et al.*, 2008 ▸) and field emitters (Wang *et al.*, 2005 ▸; Li *et al.*, 2004 ▸), among other sensor systems based on piezotronics (Wang, 2007 ▸). Many of these reports relied on anisotropic ZnO nanostructures, *e.g.* nanospikes, which can be synthesized by manifold approaches (Singh, 2010 ▸), one of which is by flame transport (Mishra *et al.*, 2013 ▸).

A fundamental understanding of functional nanomaterials requires in-depth analysis procedures to determine morphology, defects (Kienle & Simon, 2002 ▸), interfaces (Wen *et al.*, 2013 ▸), doping (Lu *et al.*, 2015 ▸), band structure (Shi *et al.*, 2012 ▸) and local deformation behaviour, as all of these aspects contribute to and affect the final device properties. In this pursuit, transmission electron microscopy (TEM) offers a variety of techniques from structure determination up to nanoscale chemical analysis. Optimized sample preparation is perhaps the most critical and challenging prerequisite for these TEM techniques, and the sample preparation must be tailored to the specific task and scientific issue (Jia *et al.*, 2011 ▸). Site-specific sectioning along selective crystal orientations is critical for structure–property investigations of anisotropic nanostructures including buried defects (Hrkac *et al.*, 2013 ▸), and strain and chemical integrity at interfaces (Abes *et al.*, 2013 ▸; Hrkac *et al.*, 2013 ▸; Huang *et al.*, 2014 ▸). In preparing these sections, filler materials, such as epoxy resins (Müller & Krumeich, 2000 ▸; Lenrick *et al.*, 2014 ▸), platinum (Giannuzzi & Stevie, 1999 ▸), carbon (Leer *et al.*, 2009 ▸; Baram & Kaplan, 2008 ▸) or aluminium oxide (Stiegler *et al.*, 2012 ▸), are often used as stabilizing matrix materials or protective coatings against ion bombardment.

Electron-transparent slices of matrices containing nano­structures are typically prepared by ion-beam etching techniques such as precision ion polishing and focused ion-beam (FIB) milling or by ultramicrotomy (Huang *et al.*, 2014 ▸; Chen *et al.*, 2004 ▸). Limiting possible modifications to the sample during the preparation procedure is critical for quality high-resolution (HR)TEM investigations. This is particularly true when dealing with very sensitive nanostructures such as wires or hollow tubes. Possible sources of damage include shrinking of epoxy resins during the solidification and curing process (Cairney & Munroe, 2001 ▸), and the introduction of residual stresses and knock-on damage (Egerton *et al.*, 2010 ▸; Bowden & Brandon, 1963 ▸) during the deposition of protective coatings or ion bombardment. Even diamond-knife ultramicrotomy can lead to mechanically damaged cross sections and increase the likelihood of coating delamination (Lipomi *et al.*, 2010 ▸). In the case of targeting special orientations, FIB preparation offers unique and diverse approaches to keep sample modifications to a minimum. Examples include a ‘direct lift-out’ procedure (Li *et al.*, 2006 ▸, 2003 ▸), and milling under small grazing angles and lower energies during the final milling steps.

Particularly when working with very fragile nanostructures, a new approach, the shadow-FIB method introduced by Welz *et al.* (2005 ▸), enables nearly artefact-free TEM specimen preparation by using the substrate as protection during heavy-ion milling. Thus, the shadow-FIB method circumvents the deposition of metal protective layers and keeps modifications and contaminations to a low level. The shadow-FIB procedure has enabled site- and orientation-specific sectioning of fragile specimens such as layered crystals (Spiecker *et al.*, 2006 ▸), organic films (Kim *et al.*, 2009 ▸; Mor *et al.*, 2014 ▸) and in particular anisotropic nanostructures (Tessarek *et al.*, 2013 ▸; Vieweg *et al.*, 2012 ▸).

Here we have prepared ZnO nanospike cross sections using the geometric shadow-FIB technique. The ZnO nanospikes were embedded in an amorphous carbon matrix by electron-beam-induced decomposition of an organic precursor. Several regions of the lamella were thinned to expose different nanospikes for HRTEM investigations. This enabled the direct observation and identification of two types of twin boundary in the ZnO nanospikes which were not observed during plan-view examination.

## 2. Experimental   

Synthesis of ZnO nanospikes was conducted following the flame-transport approach (Mishra *et al.*, 2013 ▸), in which Zn microparticles with typical diameters of 10 µm are mixed homogenously into a slurry of polyvinyl butyral (PVB) powder and ethanol. A typical weight ratio for Zn:PVB:ethanol is 1:2:6, which can be varied depending upon the requirements for morphology and specific applications. Further steps involve coating of Si substrates via a slip-casting technique and subsequent heating inside a simple muffle-type box furnace to 873 K for 1 h with a ramp rate of 100 K min^−1^. The PVB–ethanol mixture thereby acts as a sacrificial spacing layer between the particles which decomposes completely at elevated temperatures.

HRTEM was carried out on an FEI Tecnai F30 G^2^ STwin microscope (FEG, 300 kV, spherical aberration *C*_S_ = 1.2 mm) and low-resolution scanning transmission electron microscopy (STEM) imaging on a TITAN 80-300 (image corrector). Precession electron diffraction (PED) was conducted on a Philips CM 30 ST microscope (LaB_6_, 300 kV) equipped with a spinning star device (NanoMEGAS). HRTEM micrographs were obtained by tilting the individual specimens into the 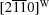 zone-axis orientation, which allowed for visualizing planar defects without superposition.

For the simulation of HRTEM micrographs and fast Fourier transforms (FFTs) the software *eMAP* (Version 1.0) by AnaliTEX (Oleynikov, 2011 ▸), the *JEMS* program package (Stadelmann, 1987 ▸) and the *Diamond* software (Version 3.2) for crystal and molecular structure visualization (Pennington, 1999 ▸) to assist with crystallographic computing were applied. Data evaluation was conducted with the Gatan Microscopy Suite *DigitalMicrograph* (Version 2.32) software.

An FEI Helios 600 dual-beam scanning electron microscope with focused ion beam and Omniprobe micromanipulators for *in situ* sample manipulation was used for TEM sample preparation. The gas injection system was equipped with precursors for platinum and carbon deposition.

## 3. Results and discussion   

### 3.1. Specimen preparation   

**Figure 1:**
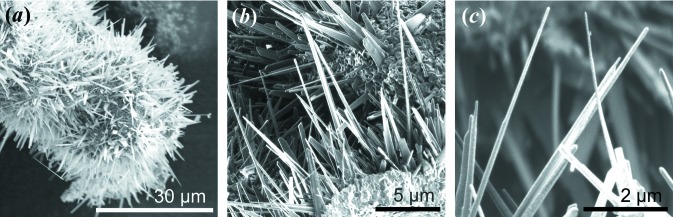
(*a*) ZnO nanospikes grown from Zn spheres via the flame-transport synthesis approach. (*b*), (*c*) The nanospikes feature a plate-like shape and show coalescence, forming spikes with multiple tips during the growth process.

After synthesis with the flame-transport approach, ZnO nanospikes were found to grow out of the Zn spheres (see the scanning electron images in Fig. 1 ▸), forming an interconnected network of particle–spike structures. Their morphology, in particular their length, can be adjusted from the nano- to the micrometre range by controlling the temperature and heating time (Mishra *et al.*, 2013 ▸). The nanospikes show tapered and plate-like morphologies with dimensions of 2–10 µm in length and *ca* 200 nm laterally. Individual nanospikes feature multiple tips, which indicate the coalescence of crystalline precipitates during growth.

**Figure 2:**
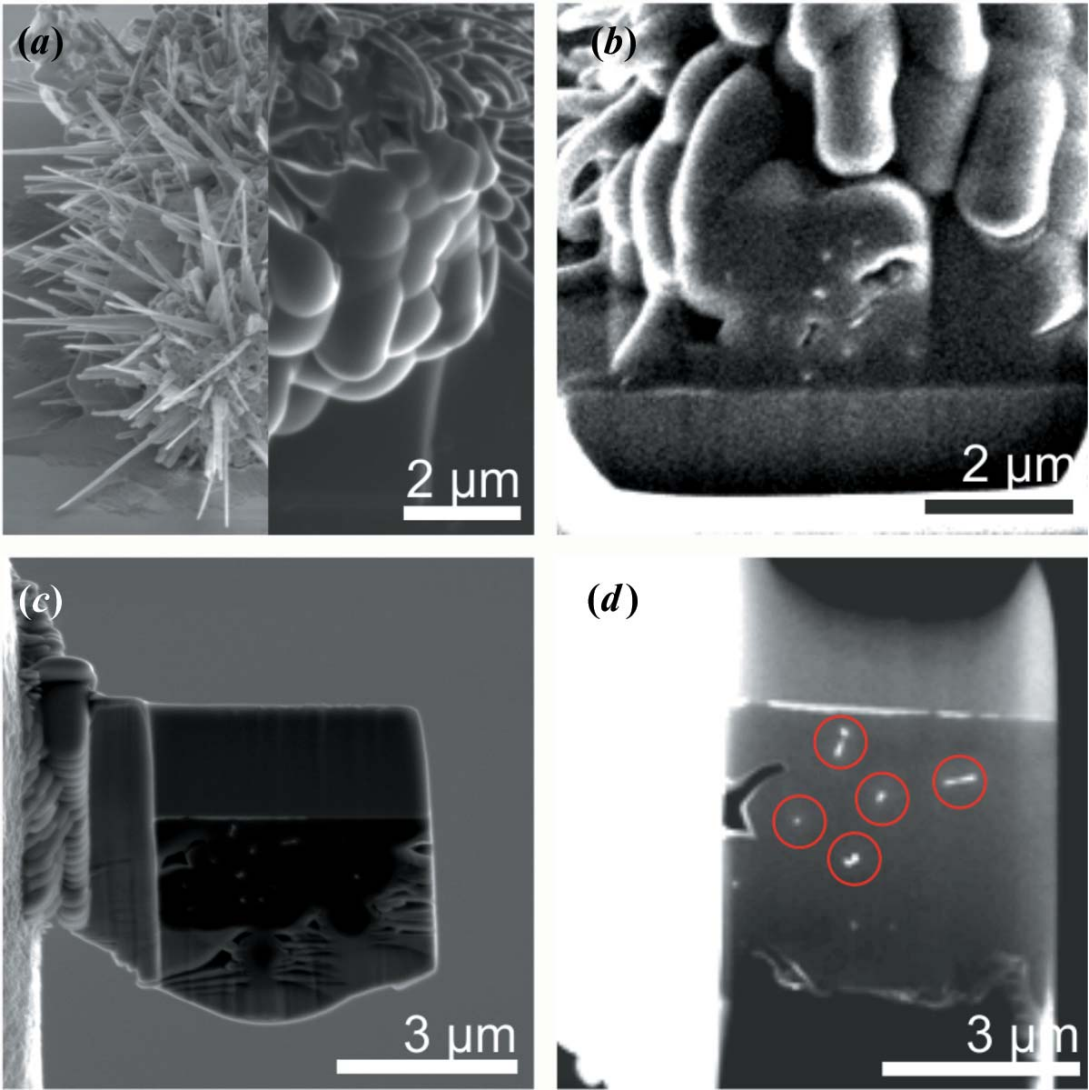
The preparation steps of ZnO nanospike cross-sections using the geometric shadow-FIB technique. (*a*) (Left) As-grown ZnO nanospikes from a Zn spherical particle. (Right) Amorphous carbon was deposited via electron-beam-induced decomposition to embed the spikes in a matrix material. (*b*) The first step of FIB trench milling and the lift-out process. (*c*) The rotated (180°) specimen was milled with a Ga ion beam using the silicon substrate as protection against severe damage to the carbon matrix. (*d*) An ion-milled lamella with electron-beam-transparent cross-section specimens (red circles).

For TEM sample preparation from freestanding ZnO nanospikes, the geometric shadow-FIB technique enabled the preparation of several cross-section specimens in one FIB lamella. The individual preparation steps are displayed in Fig. 2 ▸. Experimental problems with charging effects and the support material were circumvented by embedding the ZnO nanospikes in amorphous carbon by decomposing a naphthalene precursor with the electron beam [Fig. 2 ▸(*a*)]. To achieve conformal coating during the deposition process, a high voltage of 10 kV was applied to produce secondary electrons on all surfaces of the spikes evenly. Note that at this high voltage (the optimum is ∼2 kV) the deposition rate is much lower, so this step took about 45 min. After cutting trenches to either side [Fig. 2 ▸(*b*)] and lifting the lamella out, the sample was mounted on a needle followed by an *ex situ* 180° flip and attachment to the TEM grid, as demonstrated in Fig. 2 ▸(*c*). The top Si substrate now served as the protection layer during ion milling, since direct milling of the carbon matrix would lead to immediate destruction of the specimen. To ensure mechanical stability of the final lamella [Fig. 2 ▸(*d*)], the silicon and the edges on either side were left relatively thick.

### 3.2. Real-structure analysis   

**Figure 3:**
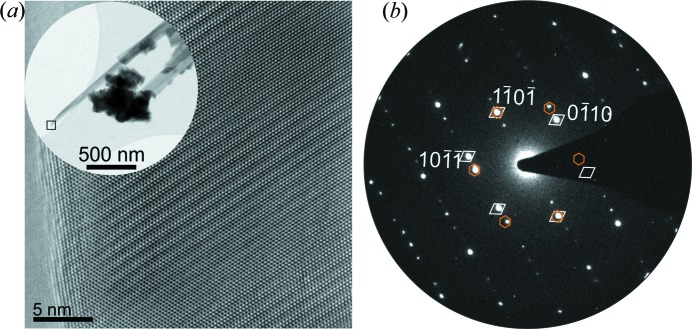
(*a*) HRTEM micrograph of a ZnO nanospike tip, showing the characteristic superposition fringe contrast as a feature of twinning. (Inset) A TEM image of the ZnO nanospike. (*b*) A precession electron diffraction pattern showing the superposition of two mirrored single-crystal patterns in 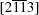 orientation. The reflections of the individual twin components are indicated by the diamond and hexagon markers.

A representative HRTEM micrograph of a ZnO nanospike tip with characteristic superposition fringe contrast is shown in Fig. 3 ▸. Previous TEM studies of ZnO nanospikes (Hrkac *et al.*, 2013 ▸; Huang *et al.*, 2009 ▸) with tapered growth morphology revealed a similar contrast phenomenon in high-resolution imaging, which was rationalized as the 2D superposition of individual 3D domains in a systematically twinned microstructure. The fringe contrast can be correlated with the superposition of two twinned domains by the PED pattern depicting the superposition of two mirrored 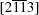 oriented patterns. The inspection of several cross-section specimens in the same lamella allowed for a detailed structural characterization using HRTEM and subsequent defect modelling.

**Figure 4:**
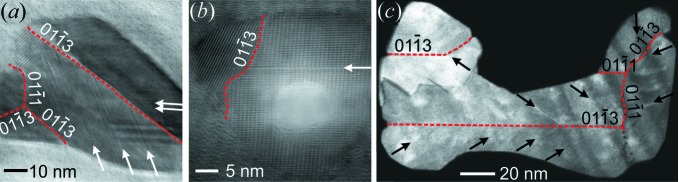
(*a*), (*b*) HRTEM and (*c*) low-resolution STEM micrographs of ZnO nanospike cross sections along their growth direction, which is 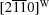. The spikes contain a high density of twinned domains (red dashed lines represent twin boundaries) and basal-plane stacking-mismatch defects (arrows). Often, twin boundaries of type 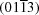 split the crystal into larger domains.

The embedded cross sections of ZnO nanospikes were analysed by HRTEM by tilting the specimens into the nearest zone axis 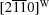, being the direction of the electron beam orthogonal on the specimen with respect to all cross sections observed. The recorded micrographs are presented in Fig. 4 ▸ and confirm the multiple twinned morphology. Two types of twin boundary are classified via the formalism [zone-axis orientation]/(twin plane) and identified by inspection of the FFTs given in Fig. 4 ▸: 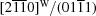 and 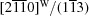. Therefore, the growth direction of these ZnO nanospikes is rationalized to be along 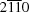 as well. For crystals with hexagonal symmetry having the wurtzite-type structure, uni­directional crystal growth along the {0001} planes is promoted under a broad range of process conditions, as this growth direction minimizes the electrostatic energy between Zn^2+^ and O^2−^ terminated surfaces. However, the introduction of planar defects parallel to the polar surfaces can decrease the surface energy and stabilize different crystal morphologies, as has already been described for nanobelts (Ding *et al.*, 2004 ▸) and whiskers (Huang *et al.*, 2008 ▸, 2009 ▸). Owing to the observed multiple twin defects, this explanation might also be applicable to the nanospikes investigated herein. A growth model for ZnO nanospikes from a partially molten Zn source including such mosaic and multiple twinning has been proposed by Huang *et al.* (2009 ▸). They described the initial twinning of condensate seeds and further coalescence and intergrowth of individual spikes, resulting in a mosaic twinned structure with a tapered shape. Very similar growth conditions are believed to apply during the flame-transport process.

A large population of basal-plane stacking faults is observed in the specimen cross sections, as indicated by arrows in Fig. 4 ▸. Note that the observation of this defect type is exclusively enabled by the cross-section view. Twinning on the 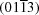 planes was observed more frequently than on the 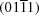 planes and often appeared to divide the nanospikes into smaller domains. In addition, shorter boundaries were observed featuring both types of twin. This is in agreement with theoretical energy calculations for twin defects in wurtzite structures (Béré & Serra, 2003 ▸), which predict the population of the 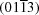 twin to be dominant over 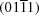.

During the HRTEM investigation, severe beam-damage effects under observation with an unmodified electron dose led to dissipation of the carbon matrix and subsequent release of the specimen into the microscope. Further, the very thin specimens suffered rapid material loss due to electron knock-on damage, as demonstrated by the large void in the centre of the cross section depicted in Fig. 4 ▸(*b*). Therefore, working with a reduced electron dose is highly recommended for cross sections embedded in a carbon matrix.

### 3.3. Structure modelling   

Models based on a supercell approach were designed for both twin boundary structures, 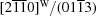 and 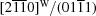, on the basis of previous studies (Hrkac *et al.*, 2013 ▸; Paulowicz *et al.*, 2015 ▸).

**Figure 5:**
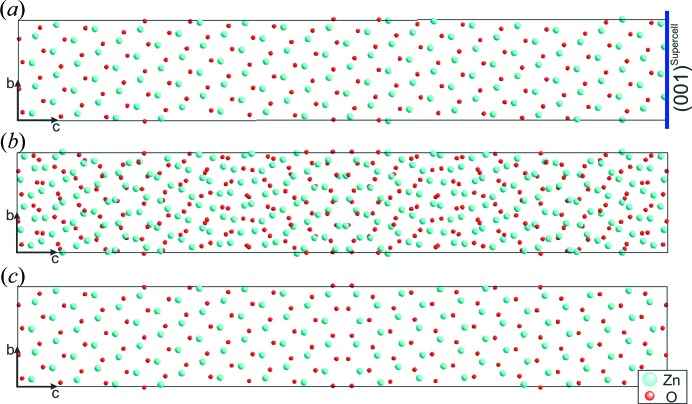
The construction of a supercell following the individual steps (i)–(iii) described in the text. (*a*) The triclinic supercell including the defect plane as one of its rectangular faces. (*b*) A superposition structure is created by mirroring the *P*1 cell at its defect plane. (*c*) The separation model containing the defect structure in the centre.

The supercell approach involved the following three steps, which are displayed in Fig. 5 ▸.

(i) The initial wurtzite-type (superscript W; hexagonal cell, *P*6_3_ *mc*) was transformed into an orthohexagonal cell. Following the conventions described by Arnold (Hahn, 2002 ▸, pp. 78–89), the unit-cell transformation of the ideal structure into a triclinic (*P*1) structure is given mathematically by
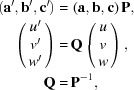
with (**a**, **b**, **c**) as the base vectors of direct space and (*u*, *v*, *w*) as the indices of a direction in direct space; primes (′) mark the parameters for the *P*1 cell. **P** and **Q** are (3×3) square matrices, linear parts of an affine transformation.

A suitable supercell for the respective defect structure is obtained by applying the transformation matrices **P**_1_ and **Q**_1_ [for the twin 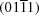], 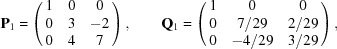
and the transformation matrices **P**_2_ and **Q**_2_ [for the twin 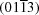], 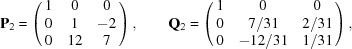
The lattice parameters for the respective supercells are *a*_1_ = 3.249, *b*_1_ = 11.835, *c*_1_ = 76.740, *a*_2_ = 3.249, *b*_2_ = 19.836 and *c*_2_ = 42.831 Å. All angles were set to 90° as the deviation from exact rectangularity is <0.1% and the resulting errors in the atom positions are marginal. The resulting supercells are rectangular and possess triclinic *P*1 symmetry.

(ii) A superposition structure (SPS) was created by adding the atomic coordinates from an unmodified supercell from step (i) to its mirrored version. The appropriate mirroring of the supercell was accomplished by the inversion of the *c* parameter, corresponding to a mirror plane at (0 0 1)^Supercell^. Note this procedure is analogous for both supercells.

(iii) A separation model is obtained by the deliberate reduction of the atoms in the respective SPS. In this manner unit cells are generated containing two single domains separated by a twin plane. To generate a good agreement between the experimental and simulated data, the separation models were adjusted in an iterative approach by shifting the atomic parameters to achieve matching contrast between simulated and experimental images.

### 3.4. Simulation   

**Figure 6:**
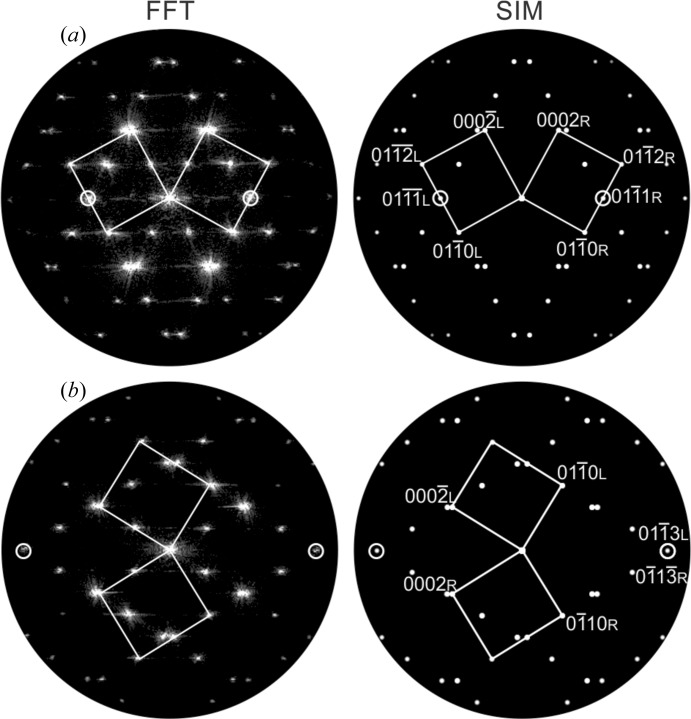
Qualitative comparisons of (left) experimental FFT images recorded at two twin boundaries and (right) simulations based on kinematic scattering using the superposition structure models. (*a*) 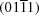 and (*b*) 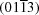. The indices L and R denote reflections from the left and right twin component, respectively.

The superposition structures [step (ii)] of each twin model were used for the simulation of electron diffraction (ED) patterns rendered possible by the translational invariance of the reciprocal lattice. The simulated ED pattern and FFT images calculated from experimental HRTEM micrographs of the two twin boundaries are compared qualitatively in Fig. 6 ▸. Both the FFT images and the simulated ED pattern from the structure models match on a qualitative basis, indicating the quality of the superposition structure. However, owing to the impact of the contrast transfer function on HRTEM micrographs, an exact match between the FFT and the pure kinematic simulation is not possible.

**Figure 7:**
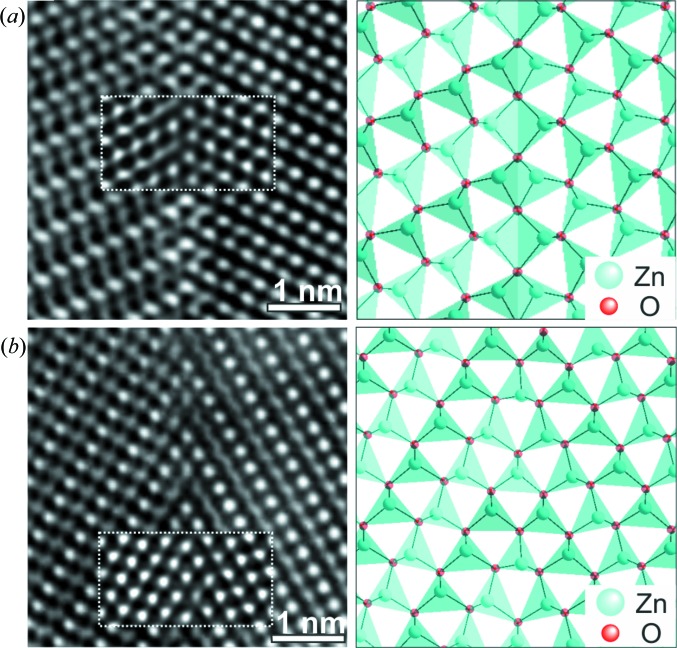
(Left) HRTEM images of the twin interfaces and (right) simulations of the inset panels for (*a*) the 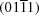 boundary and (*b*) the 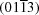 boundary using appropriate models from the supercell approach.

To approximate the atomic interface structures, the separation models were used for HRTEM simulations. The atomic positions at the interface of the separation models were adjusted iteratively to optimize the simulation pattern in agreement with the experiment. The results of this process are presented in Fig. 7 ▸, showing the HRTEM micrographs together with the simulations and interface structure models. The simulation parameters for the 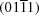 twin are defocus value Δ*f* = −24 nm and thickness *t* = 3.9 nm. The simulation parameters for the 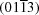 twin are Δ*f* = −58 nm and *t* = 2.6 nm. In the case of the 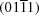 twin boundary, the model best fitting the experiment contains a single plane of oxygen atoms formed by the direct coalescence of two 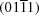 planes. This atomic configuration results in a conformation of ZnO_4_ tetrahedra that are connected by common edges and surfaces at the boundary. For such a configuration the electrostatic repulsion energies are expected to be quite large. For the 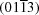 twin boundary, an atomic configuration with slightly distorted tetrahedra connected via common corners yielded excellent agreement with the experimental high-resolution contrast.

After verification with the experimental data, the designed structure models were compared with energy-minimizing computer calculations based on the work by Béré & Serra (2003 ▸). For the twin defects presented here they calculated the energetically most favourable atomic boundary structures for GaN in its wurtzite-type structure using an empirical inter­atomic potential of the Stillinger–Weber type and the quench–molecular dynamic method. The formation energies for different atomic boundary structures have been discussed for both types of twin boundary. According to this work, a related atomic boundary structure for the 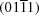 twin was calculated, exhibiting a single plane of atoms formed by the head-to-head coalescence of two 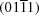 planes. This head-to-head junction possesses the highest calculated formation energy of ∼3.5 J m^−2^ in GaN, which is about 0.8 J m^−2^ larger than for boundary structures with a corrugated or head-to-tail interface. Furthermore, first-principles density-functional total-energy calculations by Yan *et al.* (2005 ▸) showed, in principle, that the same twin boundary structures are formed in ZnO as in wurtzite group III nitrides but with ∼1/3 of the GaN twin boundary energy. Hence, the above considerations can be expected to hold qualitatively true in ZnO and can explain the small number of these head-to-head 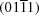 type of junctions in the nanospikes compared with the corrugated conformations observed. For some of the most common and energy-reduced twin junctions in ZnO nanowires, Shan *et al.* (2009 ▸) calculated the boundary energies to be 0.67 J m^−2^ for the head-to-tail 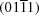 twin and 0.53 J m^−2^ for the head-to-head 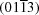 twin boundary. These numbers validate the larger population of 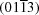 boundaries observed in the nanospikes, also acting as a structural feature in the growth process. Relaxed structure models of the latter congruent with the model presented here are reported in the literature (Ding & Wang, 2009 ▸; Shan *et al.*, 2009 ▸). Hence, we conclude that our models provide reasonable approximations for twin boundary structures of the described type in ZnO nanospikes.

**Figure 8:**
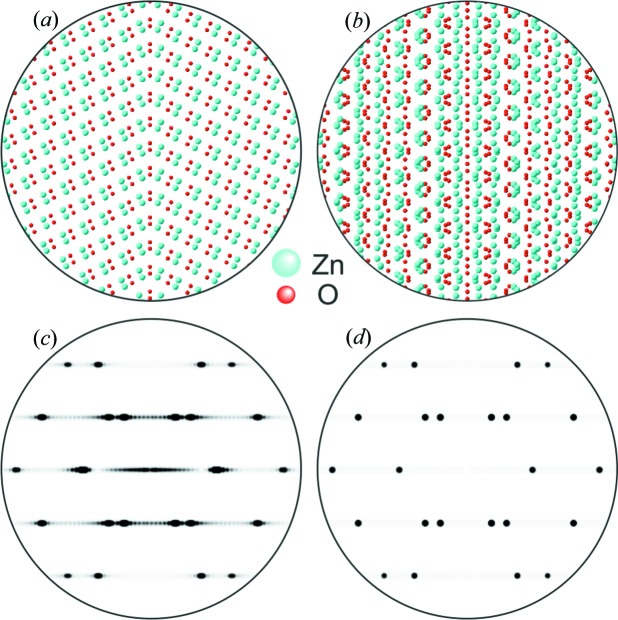
(*a*) The new model for the atomic 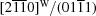 twin interface was tilted into a viewing direction congruent with 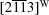. (*b*) A superposition structure was constructed by introducing mirror symmetry. (*c*), (*d*) Simulated electron diffraction data for the 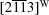 zone axis using an SPS with (*c*) and without (*d*) the twin, see text.

This direct observation and identification of two types of twin boundary in ZnO nanospikes presented herein provides complementary information to previous plan-view studies by Hrkac *et al.* (2013 ▸). Those authors presented an in-depth discussion of the origin of HRTEM superposition contrasts, as well as identification of twin planes 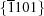 observed in the 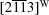 viewing direction using suitable supercell models. Since similar samples containing nanospikes grown from Zn particles were investigated in our studies, it is not surprising that the same twin planes were observed. The congruent results are demonstrated by tilting the 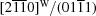 structure model into a direction equal to 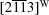 as shown in Fig. 8 ▸. In addition, an SPS is generated by introducing mirror symmetry. The tilted *P*1 model and the superposition structure are presented in Figs. 8 ▸(*a*) and 8 ▸(*b*), respectively. The simulated diffraction pattern of this SPS (space group *P*1*m*) and the SPS constructed by Hrkac *et al.* are depicted in Figs. 8 ▸(*c*) and 8 ▸(*d*) for comparison. Both simulated ED patterns depict qualitatively the same arrangement of spot pattern. The evident superstructure reflections [looking like diffuse streaks in Fig. 8 ▸(*c*)] arise because of the implementation of the single planar twin defect in the model which introduces additional lattice periodicity.

## 4. Conclusions   

In this work, the cross-sectional investigation of twin defects in ZnO nanospikes complements previous plan-view studies and more generally opens up the fundamental characterization of complex and highly anisotropic nanostructures containing structural defects. The preparation of multiple cross-section specimens of nanospikes attached to a substrate particle to achieve electron transparency was established using the shadow-FIB geometry. Intrinsic and multiple twinning was observed to be a main structural feature and is assumed to reduce the surface energy during the growth process along an energetically more unfavourable 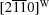 direction. Two types of twin boundary could be identified and simulated on the basis of structure models from a supercell approach. The phase-contrast simulations revealed the nature of the approximate twin boundary configurations and a direct link to plan-view investigations could be demonstrated.

In conclusion, the combination of both plan-view and cross-section analysis allows for an unambiguous determination of structural defects which are not directly accessible with a simple plan-view experiment. This combined 3D crystallographic examination approach proved to be extremely valuable and could be extended to a variety of anisotropic nanostructures.
